# Automatic Microaneurysms Detection Based on Multifeature Fusion Dictionary Learning

**DOI:** 10.1155/2017/2483137

**Published:** 2017-03-21

**Authors:** Wei Zhou, Chengdong Wu, Dali Chen, Zhenzhu Wang, Yugen Yi, Wenyou Du

**Affiliations:** ^1^Faculty of Robot Science and Engineering, Northeastern University, Shenyang, Liaoning 110004, China; ^2^College of Information Science and Engineering, Northeastern University, Shenyang, Liaoning 110004, China; ^3^School of Software, Jiangxi Normal University, Nanchang, Jiangxi 330022, China

## Abstract

Recently, microaneurysm (MA) detection has attracted a lot of attention in the medical image processing community. Since MAs can be seen as the earliest lesions in diabetic retinopathy, their detection plays a critical role in diabetic retinopathy diagnosis. In this paper, we propose a novel MA detection approach named multifeature fusion dictionary learning (MFFDL). The proposed method consists of four steps: preprocessing, candidate extraction, multifeature dictionary learning, and classification. The novelty of our proposed approach lies in incorporating the semantic relationships among multifeatures and dictionary learning into a unified framework for automatic detection of MAs. We evaluate the proposed algorithm by comparing it with the state-of-the-art approaches and the experimental results validate the effectiveness of our algorithm.

## 1. Introduction

Diabetic retinopathy (DR) is the main cause of blindness associated with diabetes [1]. The majority of people suffering from diabetes mellitus will eventually develop DR. Early diagnosis through regular screening has been shown to prevent visual loss and blindness. Color fundus photography is characterized with low-cost and patient friendliness which are a prerequisite for large scale screening [2]. However, a large number of diabetic patients need to be screened annually, which poses a huge workload for ophthalmologists. Therefore, developing an automatic DR screening system is necessary, which can not only reduce the workloads of ophthalmologists but also improve the accuracy of detection [3].

**Figure 1:**
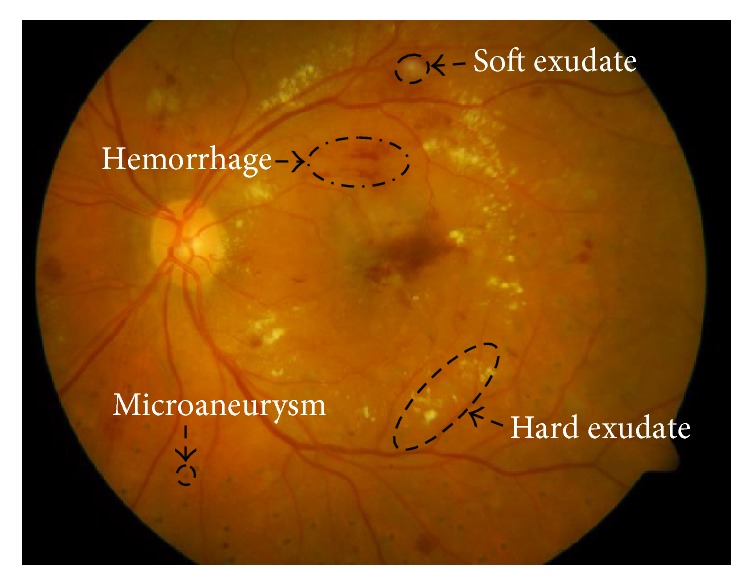
Fundus image containing lesions.

Signs of DR contain red lesions such as microaneurysms and hemorrhages, yellowish or bright spots such as hard and soft exudates (see Figure 1). In this paper, we mainly focus on the detection of microaneurysms, which present at the earliest stage of DR and remain in the development of the disease [4]. Therefore, microaneurysms detection is necessary and vital in public DR screening programs.

Numerous approaches have been proposed for microaneurysm detection, which always involve three fundamental processing phases: preprocessing with the normalization of the images, candidate extraction which is to locate all possible MA candidates, and MA classification based on features computed on each candidate [5].

The earliest paper based on MA detection was proposed by Baudoin et al. [6] using a mathematical morphology approach to detect the microaneurysms in fluorescein angiograms. After that, two variants of morphological top-hat transformation methods for segmenting MAs within fluorescein angiograms were developed by Spencer et al. [7] and Frame et al. [8]. Although using the fluorescein angiograms can improve the contrast between the fundus and their background, the usage of intravenous contrast agents is dangerous and even associated with mortality [9], which cannot be widely used in public DR screening programs. Besides, mathematical morphology approaches mainly rely on the choosing of structuring elements, which may increase false positives or decrease true positives when changing their size and shape.

Several approaches based on machine learning have been proposed to distinguish the MA from the non-MA. Niemeijer et al. [10] presented a red lesion detection method based on morphological top-hat transform and used the *k*-nearest neighbor algorithm as pixel classification. In their method, a series of features including the features provided by Spencer et al. and Frame et al. [7, 8] and some new features were used for characterizing object candidates. Sánchez et al. [11] suggested a combination of Gaussian mixture model and a logistic regression classification to classify MAs at pixel level. Since pixel level classification methods are mainly based on medical experts labeling at pixel level, they are unsuitable for dealing with too many large size fundus images [9]. Akram et al. [12] extracted a set of features containing shape, intensity, color, and statistical properties for each candidate, and then a hybrid classifier was used to improve the accuracy of classification. Besides, artificial neural network (ANN) [13] and convolution neural network (CNN) [14] were also applied to detect the lesions in fundus images. However, these methods are not suitable for a large number of training samples as the training time will be long.

Apart from the above-mentioned MA detection approaches, some MA detection algorithms are based on template matching using this fact that the intensity of MA exhibits a Gaussian shape [15, 16]. Quellec et al. [15] proposed a method by using wavelet image decomposition as template for MA detection. The problem of illumination variations or high-frequency noise can be avoided effectively in this approach. Zhang et al. [16] employed Multiscale Gaussian Correlation Coefficients (MSCF) to detect MA. In their model, MA candidates can be detected by using a nonlinear filter with five varying Gaussian kernels to the input image. Generally, the main challenge of template matching approach is how to design an accurate template to match the MA.

Nowadays, sparse representation-based classification (SRC) has achieved promising outcomes in classification. Inspired by SRC, Zhang et al. [17] proposed MA detection method, which combined the dictionary learning (DL) with SRC. Firstly, Multiscale Gaussian Correlation Coefficients filtering was used to locate all the possible candidates, and then these candidates were classified by SRC. After that, Javidi et al. [18] combined discriminative dictionary learning with sparse representation for MA detection. Firstly, Morlet wavelet algorithm was applied to detect MA candidates. Next, two discriminative dictionaries containing MA and non-MA dictionaries were learned with the aim of distinguishing the MAs from non-MAs. Finally, MAs are classified by using the two learned dictionaries.

However, the above-mentioned approaches [17, 18] depended heavily on original grayscale feature dictionary for MA detection, and since there is a large variability in color, luminosity, and contrast both within and between retinal images, using single grayscale feature will affect the performance of MA detection. In this paper, MA detection approach based on multifeature fusion dictionary learning has been developed. The learned dictionary not only takes the semantic relationships among the multifeature into consideration but also adapts to the content of image. Hence, it is estimated to outperform the dictionary constructed by a single grayscale feature. In our proposed approach, first of all, preprocessing is adopted to reduce uneven illumination, poor contrast, and noise. Secondly, MSCF (Multiscale Correlation Filter) is applied to identify all possible MA candidates from the fundus images. Then, MA image patches and non-MA image patches can be extracted from these candidates. Next, a series of features are used to characterize these image patches forming multifeature dictionary. Finally, with the learned dictionary, the class label of every query candidate identified in the previous step can be determined by computing the total reconstruction error of multifeature for each class.

The remainder of this paper is organized as follows: a brief review of the concept of sparse representation classification and multifeature fusion dictionary learning is presented in Section 2. A description of the way multifeature fusion dictionary learning for MA detection is presented in Section 3. Experimental results are presented in Section 4. Finally, the conclusion is given in Section 5.

## 2. Preliminaries

In recent years, sparse representation (or sparse coding) and dictionary learning have attracted wide attention and been successfully used in signal and image and video processing and biometric applications [19]. In this section, we firstly give the essential concept of sparse representation classification. Then, we give a brief introduction about the multifeature fusion dictionary learning.

### 2.1. Sparse Representation Classification

Sparse Representation-based Classification (SRC) [20] has attracted a lot of attention for its applications to various tasks, especially in face recognition proposed by Wright et al. In SRC, it is assumed that the query sample can be regarded as a linear combination of all the training samples. Suppose that there are *K* classes of subjects, and let *A* = [*A*_1_, *A*_2_,…, *A*_*K*_] ∈ *R*^*D*×*N*^ denote the set of training samples. Here, we regard it as a dictionary, where *A*_*i*_ = [*a*_*i*1_, *a*_*i*2_,…, *a*_*iN*_*i*__] ∈ *R*^*D*×*N*_*i*_^ is the subset of training samples from class *i*, in which each column vector *a*_*ij*_ represents the *j*th sample of the *i*th class, *D* is the dimension of each training sample, and *N*_*i*_ is the number of training samples of *i*th class (*N* = ∑_*i*=1_^*K*^*N*_*i*_). A query sample *y* ∈ *R*^*D*^ can be represented by training samples of all classes as follows:

(1) $$\begin{array}{c} y=A\alpha +e, \end{array}$$

where *α* is the representation coefficient vector of *y* and *e* is the representation error.

There is a basic assumption that samples of a specific subject lie in a linear subspace [20]. With this assumption, a query image is expected to be well represented as a liner combination of just those training samples from the same class. The sparse linear representation model seeks to solve the following optimal problem:

(2) $$\begin{array}{c} \hat{\alpha }={\underset{\alpha }{arg\;min}\;\left\|y-A\alpha \right\|}_{2}+\lambda {\left\|\;\alpha \;\right\|}_{0}, \end{array}$$

where ‖·‖_0_ denotes the *l*_0_-norm, simply counting the number of nonzeros entries in *α*, and *λ* ≥ 0 is a tradeoff parameter between the two terms. Since (2) is NP-hard problem [20], most of spares representation researches [20–22] employ the *l*_1_-norm constraint to relax the *l*_0_-norm constraint. Therefore, the original equation (2) can be denoted by the following *l*_1_-norm minimization:

(3) α^=arg minα y−Aα2+λα1.

Here, there are two terms in (3), the first term is the reconstruction error, and the second term is a sparsity measurement. ‖·‖_1_ denotes the *l*_1_-norm that is simply the sum of the absolute values of the columns.

Since optimization problem (3) is convex, some well implemented toolboxes such as NESTA [22] can be used to solve it. Having obtained the optional solution $\hat{\alpha }$, the class label of *y* can be acquired by the following criterion:

(4) labely=arg mini=1,…,K y−Aiα^i2,

where ${\hat{\alpha }}_{i}$ is the component of $\hat{\alpha }$ restricted on class *i*; that is to say, the coefficients *α* associated with class *i* can be retained and the others are 0.

Even though the sparse representation classification model described in [20] achieved quite good performance, moreover, two drawbacks are listed as below: on one hand, since the raw training samples contain noise, directly using them as the dictionary may reduce the effectiveness of classification. On the other hand, SRC just uses raw image pixel as intuitive feature, which is not robust subject to the lighting conditions and other small changes [23].

### 2.2. Multifeature Fusion Dictionary Learning

In order to overcome the above-mentioned drawbacks, sparse representation based on multifeature fusion (MFF) has been introduced which combines the semantic relationships among multifeatures and improves the classification performance.

Here, tensor algebra is adopted to achieve multifeature fusion. The computation and notation used in this paper mainly follow [24, 25]. Particularly, given a high-order tensor *χ* ∈ *R*^*I*_1_×*I*_2_×⋯×*I*_*K*_^, *K* is said to be its order number and the dimension of the *k*th order is *I*_*k*_. Suppose there are *N* observations and each of them can be represented as a *K* order tensor, that is, *χ*_*i*_ ∈ *R*^*I*_1_×*I*_2_×⋯×*I*_*K*_^,  *i* = 1,2,…, *N*. *χ*_×*k*_*U* ∈ *R*^*I*_1_×*I*_2_×⋯×*I*_*k*−1_×*J*×*I*_*k*+1_×⋯×*I*_*K*_^ is the *k*-mode product of a *K*th order tensor *χ* ∈ *R*^*I*_1_×*I*_2_×⋯×*I*_*k*−1_×*I*_*k*_×*I*_*k*+1_×⋯×*I*_*K*_^ by matrix *U* ∈ *R*^*J*×*I*_*k*_^.

In MFF, suppose we have already learned *K* feature dictionaries arranging them to a tensorial representation *D* ∈ *R*^*p*×*d*×*K*^. The *k*th feature dictionary can be expressed as *D*_*k*_ ∈ *R*^*p*×*d*^. Given a query image *e* = [*e*_1_,…, *e*_*i*_,…, *e*_*K*_] ∈ *R*^*p*×*K*^, in which *e*_*k*_ represents the *k*th feature, the multifeature fusion object function is formulated as

(5) β1,…,βK=arg minβ1,…,βK ∑k=1Kek−DkβkF2+λΦβ1,…,βK,

where *D*^*k*^ is the *k*th slice of *D* along the third mode and *β*_*k*_ are the corresponding coefficients of *e*_*k*_ over *D*^*k*^. *λ* is the scalar parameter and a more strict group-level sparsity constraint Φ(·) [26] is imposed on the coefficients. For each class, just the atoms from the same class can be used for representing query sample.

In order to make full use of the relationships between these dictionaries belonging to *D* and lower the computational burden, the tensor representation dictionary *Q* ∈ *R*^*p*×*d*×*M*^ can be regarded as a core dictionary (*M* is the number of fused dimensions, *K* > *M* or *K* ≫ *M*) and linearly transformed from *D* in terms of a transform matrix *W* ∈ *R*^*K*×*M*^, such that *Q* = *D*_×3_*W*^*T*^. Besides, all the features of each query are needed to be calculated by (5). However, this process is time-consuming. In order to improve the effectiveness of computation, we take an alternative solution by employing the fusion matrix *W* directly on query image *e* by *e* × *W* and obtain a compact representation *y* ∈ *R*^*p*×*M*^. An alternative object function based on (5) can be rewritten as below:

(6) β1,…,βM=arg minβ1,…,βM ∑t=1Myt−QtβtF2+λΦβ1,…,βM,

where *y*_*t*_ represents the *t*th feature of fused datum *y* and  *Q*_*t*_ is the *t*th subdictionary of core dictionary *Q*.

Now, how to learn the fusion matrix *W* and how to learn the core dictionary *Q* become critical problems for solving coefficient matrix [*β*_1_,…, *β*_*M*_]. Here, a two-step manner can be used to solve this problem, that is, learning fusion matrix *W* firstly and then learning the core dictionary *Q*.

Although multifeature extraction can bring much valuable information which improves the performance of classification, more features also bring some redundancy accordingly. For keeping balance between them, multifeature fusion can be regarded as a good manner to solve the above-mentioned problem. Here, Fisher criterion [27] which maximizes the between-class scatter and minimizes the within-class scatter simultaneously is used to make the fused features have more discrimination. Suppose there are *C* classes training samples with *K* features and each class has *N*_*c*_ samples. Let *X*_*i*_ ∈ *R*^*p*×*K*^ denote the *i*th sample for *i* = 1,…, *N*. The fusion matrix *W* can be derived as below:

(7) W=arg maxW SbSw,

where

(8) Sb=∑c=1CNcX−c−X−TX−c−X−,Sw=∑c=1C ∑Xi∈cXi−X−cTXi−X−c,

where ${\overset{-}{X}}_{c}$ is the mean vector of the *c*th class and $\overset{-}{X}$ is the mean vector of the whole dataset.

The above optimization problem can be regarded as the generalized eigenvalue problem below [27]:

(9) $$\begin{array}{c} S^{b}\varphi =\lambda S^{w}\varphi , \end{array}$$

where *λ* is the generalized eigenvalue and the vector *φ*_*m*_ is the corresponding eigenvector which is one of the columns of the Fisher transform matrix *W* = [*φ*_1_,…, *φ*_*m*_,…, *φ*_*M*_].

After obtaining the target fusion matrix *W*,  *K* features can be fused into more compact and more discriminative *M* features and the fused training data *y*_*i*_ = *X*_*i*_ × *W* ∈ *R*^*p*×*M*^ can be obtained accordingly (*K* > *M* or *K* ≫ *M*,  *i* = 1,…, *N*). With the fused training data *y*, the core dictionary *Q* ∈ *R*^*d*×*p*×*M*^ can be divided into *M* × *C* subdictionaries, where *C* is the number of classes and *Q*_*m*_^*c*^ denotes the *c*th class individual and the *m*th feature. After that, *K*-SVD [28] is applied to learn each subdictionary for each feature. It can be seen as an iterative method that alternates between sparse coding of the examples based on the current dictionary and a process of updating the dictionary atoms to better fit the data.

In the test stage, given a query sample *q* ∈ *R*^*p*×*K*^, apply the fusion matrix *W* to it and derive the fused result *y* = *qW* ∈ *R*^*p*×*M*^. Here, local sparse coding based method [29] is adopted for classification and the sparse representation coefficient can be achieved by solving a least square problem. After obtaining the sparse representation coefficient matrix [*β*_1_,…, *β*_*M*_], the corresponding reconstruction error of each feature subdictionary can be calculated as below:

(10) errorcm=minβ ym−Qmcβ22,m=1,…,M,  c=1,…,C.

Summarize the error of all *M* features of each class and get the final class of the query sample *q* as:

(11) labelq=arg minc ∑m=1Merrorcm.

## 3. Proposed Method

**Figure 2:**
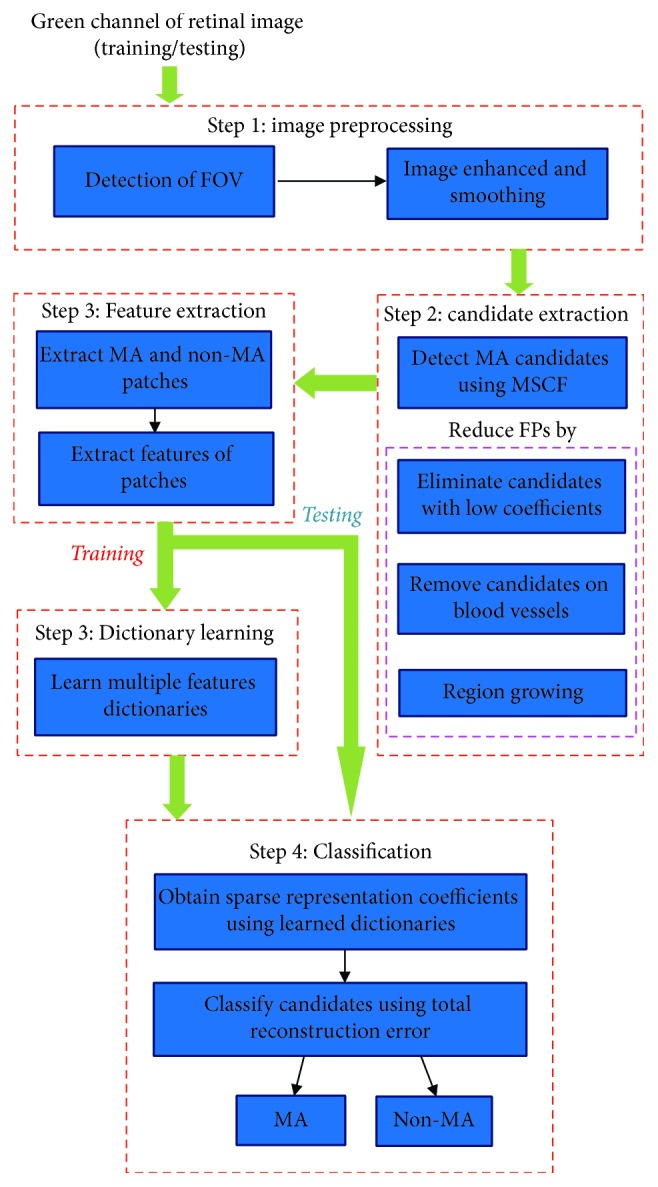
The block diagram of our proposed approach.

In this section, we will introduce the proposed multifeature discrimination dictionary learning for MA detection. It consists of the following four steps: preprocessing, candidate extraction, multifeature dictionary construction, and classification. In the first step, firstly, we extract a region of interest in the retinal image with the aim of reducing processing time. Also, the contrast and intensity between the background and MAs are enhanced for making MA more visible. In the second step, all the possible MA candidates are extracted using Multiscale Gaussian Correlation Filtering. At the same time, some operations are used to remove the FPs for reducing the number of non-MA candidates. Next, extract a series of image patches from the above obtained candidates and then multiple features are used for characterizing these image patches forming multifeature dictionaries. In the last step, with the obtained multifeature dictionaries, true MAs can be identified from the whole candidates. Each of these steps will be discussed in detail in the following sections. The workflow diagram of our proposed approach is shown in Figure 2.

### 3.1. Image Preprocessing

#### 3.1.1. Field of View (FOV)

**Figure 3:**
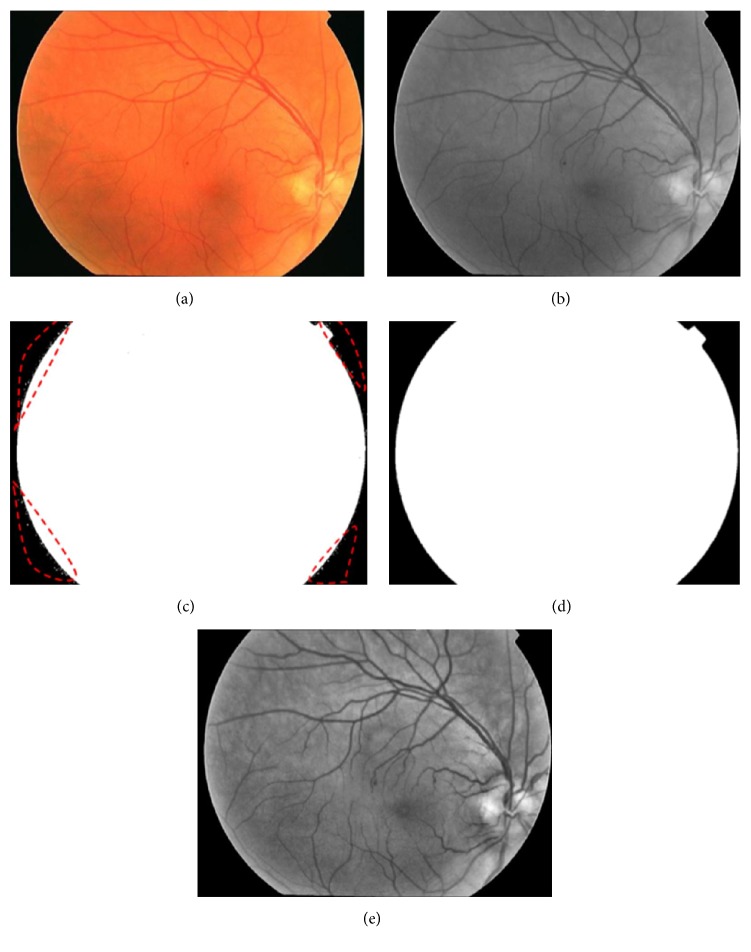
Preprocessing. (a) Original retinal image; (b) green channel of (a); (c) coarse binary FOV mask image; (d) the final FOV image; (e) result of preprocessing.

The field of view (FOV) can be regarded as the circle region containing the eye fundus information. And the pixels located just in FOV are useful for our proposed MA detection approach. Therefore, it is necessary to mask the pixels outside of the FOV and carry out our proposed MA detection method on FOV image. In this paper, we employ two-level hierarchical architecture for FOV extraction. In the first level (coarse level), we use Otsu threshold algorithm [30] to green channel of original image (see Figure 3(b)) to obtain the coarse binary FOV mask image (see Figure 3(c)). However, in coarse binary FOV mask image, some pixels within red circles (see Figure 3(c)) are misclassified as FOV. In order to solve this issue, in the second level, we adopt morphological opening and closing operations with the disc-shaped structuring element of size 2 to remove them from the coarse binary FOV mask image. Finally, the binary FOV mask image can be obtained (see Figure 3(d)). With the obtained FOV mask image, the ROI of retinal image can be acquired by cropping the image with its mask.

#### 3.1.2. Contrast Enhancement and Image Smoothing

The large luminosity, poor contrast, and noise always occur in retinal fundus images [10], which affect seriously the diagnostic process of DR and the automatic lesions detection, especially for MA. In order to address these problems and make a suitable image for MA detection, first is extracting the green channel of original image, in which the MAs have the higher contrast with their background. After that, contrast limited adaptive histogram equalization (CLAHE) [31] method is applied to the green-channel image for making the hidden features more visible. At the end, Gaussian smoothing filter with a width of 5 and a standard deviation of 1 is also incorporated to the above obtained enhanced image for reducing the effect of noise further, and the preprocessing result is shown in Figure 3(e).

### 3.2. Candidate Extraction

A candidate extraction method based on multiscale correlation filtering [16] proposed by Zhang et al. is applied to extract all the possible MA candidates. The details of this method are reviewed as below.

**Figure 4:**
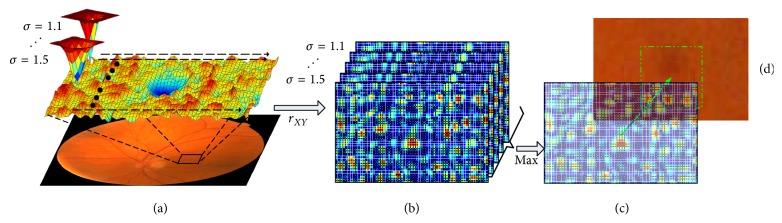
Locating MA candidates using MSCF method.

Firstly, a nonlinear filter with five different Gaussian kernels ranging from 1.1 to 1.5 with an interval of 0.1 (see Figure 4(a)) is used for calculating correlation coefficients of each pixel. Here, we denote Gaussian function and the gray distribution of MA by the variables of *X* and *Y*, respectively. The correlation coefficient can be defined as follows:

(12) $$\begin{array}{c} r_{XY}=\frac{\sum_{m}\sum_{n}\left(X_{mn}-\overset{-}{X}\right)\left(Y_{mn}-\overset{-}{Y}\right)}{\sqrt{\left(\sum_{m}\sum_{n}{\left(X_{mn}-\overset{-}{X}\right)}^{2}\right)\left(\sum_{m}\sum_{n}{\left(Y_{mn}-\overset{-}{Y}\right)}^{2}\right)}}, \end{array}$$

where $\overset{-}{X}$ and $\overset{-}{Y}$ are the mean values of *X* and *Y* and the values of correlation coefficient range from 0 to 1.

The maximum coefficient at each pixel location among the five responses (Figure 4(b)) is selected to form the final response (Figures 4(c) and 4(d)).

**Figure 5:**
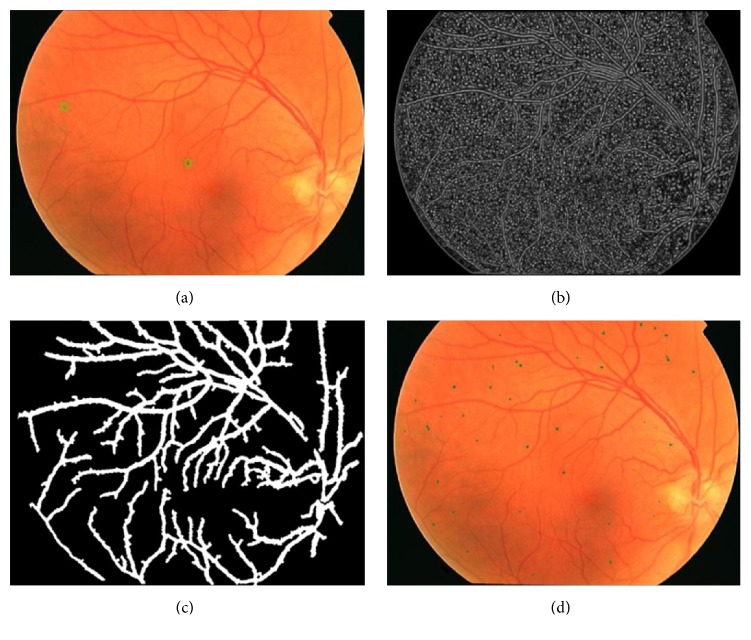
The process of candidate extraction; (a) the retinal image with annotated microaneurysms; (b) the final response of multiscale correlation filtering; (c) the output of its blood vessel map; (d) presentation of MA candidates after region growing.

Secondly, in order to reduce the number of microaneurysm candidates in final response, a threshold which ranges from 0.1 to 0.9 with an interval of 0.1 is applied to eliminate the candidates with low coefficients. Since the MAs do not appear on the vasculature, any candidates on the vasculature need to be removed [32] (the vasculature map is shown in Figure 5(c)). In addition, the size and shape of MAs are not representing the true MAs during this process and region growing [10] is used for determining their precise sizes. In the region growing, green-channel image *I*_green_ and the background image *I*_bg_ can be obtained by applying mean filter to *I*_green_. An adaptive threshold *t* based on the dynamics is given by

(13) $$\begin{array}{c} t=I_{darkest}-\beta \cdot \left(\;I_{darkest}-i_{bg}\;\right), \end{array}$$

where *I*_darkest_ denotes the lowest intensity in *I*_green_ and  *β* is a constant value ranging from 0 to 1, which is set to 0.5 here.

Region growing starts from the point of *I*_darkest_ in each candidate region and continues until no more connected pixels are higher than threshold. Considering the size of MA is less than 120 pixels [16], if the area of a region is larger than 120 pixels, it will be discarded. Finally, the remaining candidates from region growing can be regarded as the final MA candidate regions (Figure 5(d) shows overlaying region growing maps on the original image). A systematic overview of candidate extraction is shown in Figure 5.

### 3.3. MA Detection Using Multifeature Fusion Dictionary Learning

In this section, we first extract eight types of features for each candidate. Then, multifeature fusion dictionary can be learned based on all the possible MA candidates including MAs and non-MAs detected in the stage of candidate extraction. Finally, the true MAs can be classified by the learned dictionary.

#### 3.3.1. Extract MA and Non-MA Patches

**Figure 6:**
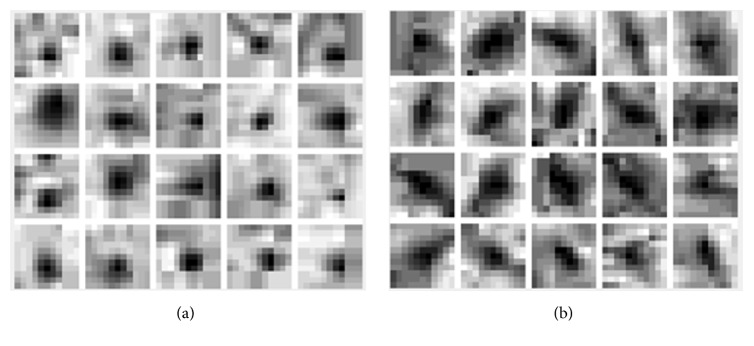
MA and non-MA training patches. (a) MA training patches; (b) non-MA training patches.

Generally, there are two categories in MA candidates including MA and non-MA. With these candidates, two classes of training samples can be constructed. The detected candidates marked by experts (the ground truth provided by ROC followed by clinicians) as MA are used for forming MA training samples. At the same time, we take the detected candidates not marked as MA as the non-MA training samples. To do this, 11 × 11 patch that covers MA or a non-MA at its center is extracted, and then these extracted patches are converted into unit column vectors by *l*_2_-norm. Figures 6(a) and 6(b) depict some selected MA training patches and non-MA training patches, respectively.

#### 3.3.2. Features Extraction of Patches and Multifeature Fusion Dictionary Learning

**Figure 7:**
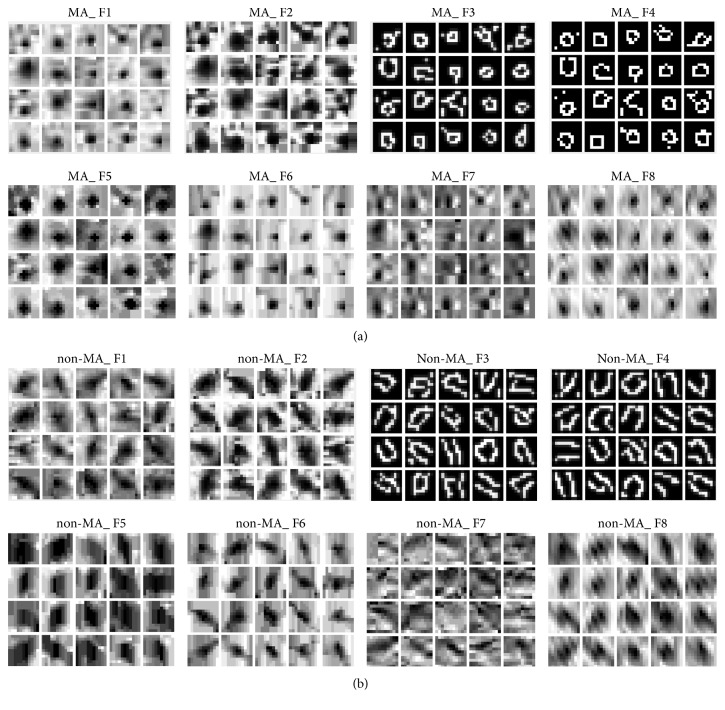
Eight features of MA and non-MA patches. (a) The features of MA patches; (b) the features of non-MA patches.

After obtaining the MA and non-MA training patches, we extract eight features for each of them including original grayscale image (coded as F1), the image after histogram equalization (coded as F2), edge image of original image by Canny operator (coded as F3), edge image of histogram equalized image by Canny operator [33] (coded as F4), morphological close operator image of original image (coded as F5), morphological open operator image of original image (coded as F6), gradient image of original image (coded as F7), and wavelet denoising image of original image (coded as F8), as illustrated in Figure 7.

With the above obtained multifeature training samples containing MA patches and non-MA patches, we can obtain a tensorial representation dictionary *D* ∈ *R*^*p*×*d*×*K*^. Here, let *p* = 121 denote the dimension of extracted image patches. We take *K* = 8 features in this paper. Based on these extracted multifeature training samples, fusion matrix *W* can be learned by using (7), which is more discriminative for better classification and more compact for efficient computation. After obtaining the fusion matrix *W*, the multifeature subdictionary *B*_*m*_^*c*^ can be acquired by *Q* = *D*_×3_*W*^*T*^ (*c* represents the class of sample and we set *c* = 1 for MAs and *c* = 2 for non-MAs, *m* denotes the number of fused features. Finally, K-SVD is used to iteratively update the multifeature subdictionary and the corresponding sparses coding until (6) converges.

#### 3.3.3. Classification

Once the multifeature discriminate subdictionaries *B*_*m*_^*c*^ and fusion matrix *W* are obtained, the label of query sample *X* ∈ *R*^*p*×*K*^ can be determined by the following three steps: first of all, apply fusion matrix *W* to it and obtain the fused datum *Y* = *XW* ∈ *R*^*p*×*M*^. Secondly, summarize the reconstruction error of all *M* features of each class as given in (10). Finally, the decision is ruled in favor of the class with the lowest reconstruction error accumulated over all *M* features as given in (11).

## 4. Experimental Results and Analysis

### 4.1. Dataset

Retinopathy Online Challenge (ROC) [34] database includes 100 (50 train samples and 50 test samples) digital color fundus photographs which were selected from a large dataset (150,000 images) in a diabetic retinopathy screening program. These photographs were obtained by using different types of camera including TopconNW 100, NW 200, or Canon CR5-45NM nonmydriatic cameras with three different sizes of field of view (FOV), and the details are listed in Table 1.

In the past, the Retinopathy Online Challenge (ROC) organization provided a way for researchers to evaluate their methods on the test images, but now this competition website is inactive [18]. It is impossible to evaluate our method on the test images. So, in our model, we just employ 50 training samples to train and verify the effectiveness of our proposed method. In the training set, there are 37 digital color fundus photographs including a total of 336 microaneurysms, and correspondingly no microaneurysms are identified in the remaining 13 images.

### 4.2. Evaluation Criteria

In our model, we choose Free-response Receiver Operating Characteristic (FROC) curve to verify the effectiveness of our proposed method. FROC curve plots the sensitivity against the average number of false positives per image (FPPI). Two evaluation mechanisms are given by the formulas in

(14) Sensitivity=True  positiveTrue  positive+False  negative,FPPI=False  positiveTotal  number  of  images,

where True positive (TP) is the number of MAs that are correctly identified; False negative (FN) is the number of those incorrectly found as non-MAs; False positive (FP) is the number of those incorrectly found as MAs.

Besides the above evaluations, we also employ precision which is the percentage of detected pixels that are actually MAs as another parameter to evaluate the effectiveness of multifeature fusion. The precision can be calculated through

(15) $$\begin{array}{c} Precision=\frac{True\;\;positive}{True\;\;positive+False\;\;positive}. \end{array}$$

Indeed MA and non-MA training samples are based on the ophthalmologist's marking, and some wrong markings may exist in provided ground truth, which will reduce the overall performance of the training and testing. In this paper, the usage of ground truth provided by ROC follows standard procedures (followed by clinicians) that ensure that all are playing with the same cards. Our proposed algorithm is being affected (the same as others) [16]. Furthermore, dictionary learning method can be regarded as a good way by automatically selecting some suitable dictionary atoms for relieving this problem. Its research and the effectiveness verification will be discussed in our future work.

### 4.3. MA Detection Results

In ROC training dataset, there are 50 fundus images in total. We randomly select 30 color fundus images for training the dictionaries and the remaining are used for testing. This process is repeated 4 times and the average result is regarded as our final result. In preprocessing stage, after extracting FOV of fundus images and enhancing contrast by using CLAEH method, all green-channel images of original fundus images are resized to the resolution of 850 × 850 pixels by bicubic interpolation. Next, a nonlinear filter with varying Gaussian kernels ranging from 1.1 to 1.5 is used for detecting all the possible MA candidates. Then, MA and non-MA image patches can be extracted based on these candidates. Also, eight types of features are used for characterizing each image patch and multiple features dictionaries can be learned accordingly. Finally, the candidates identified in the above steps can be classified using (6), (10), and (11).

The candidate extraction threshold *r*_*XY*_ is an important parameter in MSCF, which affects the performance of algorithm. In this paper, different thresholds are employed to create the FROC curve. After an analysis of the FROC curve, the optimal value of this parameter is equivalent to 0.6. Using this parameter value, the candidate detection method can achieve a sensitivity of 47.71% with a FPPI of 44.13.

**Figure 8:**
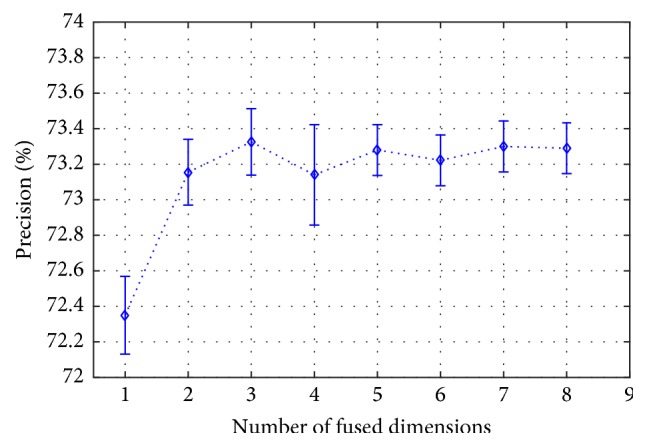
The average precision rates (%) under varying fused dimension *M*.

In order to validate the effectiveness of multifeature fusion, in our first experiment, we extract *K* features ranging from 1 to 8 based on training set and testing set. The performance is evaluated by using mean precision with standard deviation. The experimental results are plotted in Figure 8.

As shown by the results, we can see that when *M* is small, the precision is relatively low because the intrinsic relationship of different features is not fully exploited. With the increasing of *M*, more information is incorporated to dictionary learning for achieving good performance, which makes the precision keep growing and gradually become stable. According to Figure 8, we can see that that the best performance of the proposed model can be obtained with parameter *M* as three. Specifically, we set *M* = 3 to our second experiment.

**Figure 9:**
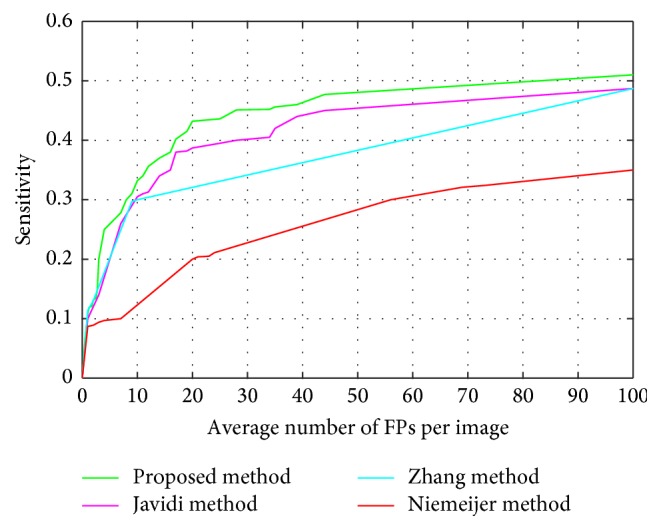
The FROC curves of the proposed method compared with the state-of-the-art methods using 30 ROC training images.

In our second experiment, we will verify the effectiveness of the proposed method by comparing it with other state-of-the-art methods [10, 17, 18] on the ROC training dataset shown in Figure 9.

As can be seen, the proposed method outperforms other methods at the same FPPI and yields a higher sensitivity.  Table 2 depicts the sensitivity at seven fixed FPPI (1, 2, 4, 8, 12, 16, and 20) and the average sensitivity of all methods derived from FROC curves in Figure 9.

According to Table 2, the proposed method shows better performance and detects more MAs at the same FPPI points. Besides, the average sensitivity of our proposed method is 0.285, which is better than other methods. The good performance is due to the following two points: on one hand, image contrast enhancement and MSCF are adopted to detect all the possible MA candidates. Since more candidates also bring in more burdens for classification, on the other hand, multifeature fusion dictionary learning is combined to our model for relaxing this burden and improving the performance of MA classification. Besides, from Table 2, we also can see that the average scores of all algorithms are relatively low. This may happen because of the quality of the images which are JPEG compressed causing some MAs to be too small or too blurred to be seen with the naked eye [16]. Even so, our algorithm is still significantly superior to the other algorithms.

## 5. Conclusion and Future Work

In this paper, we present a novel algorithm, namely, automatic microaneurysm detection based on multifeature fusion dictionary learning in diabetic retinopathy, which explicitly integrates multiple features and dictionary learning into a unified framework. Our proposed method consists of the following four steps. The first step is related to preprocessing. Next, all the possible MA candidates can be detected using MSCF. Then, extract MA and non-MA image patches. Also, multiple features are used for characterizing these image patches forming multifeature dictionary. The last step involves the true MAs classification using multifeature fusion dictionary learning method. The experiments are carried out on the standard and public available Retinopathy Online Challenge (ROC) training datasets. The experimental results indicate that our proposed method in MA detection has the better average sensitivity compared to the state-of-the-art methods.

The future work includes the following issues: firstly, our proposed model does not take the evaluating severity DR into consideration, a possible future work lies in defining the degree of DR severity. Besides, applying our proposed framework to other lesions' detection is also another interesting topic for future study.

## Figures and Tables

**Table 1:** The different types of images in the ROC training set.

	Resolution	Coverage of the retina	Number in training set
Type 1	768 × 576	45	22
Type 2	1058 × 1061	45	3
Type 3	1389 × 1383	45	25

**Table 2:** Sensitivities of different methods at various false positive points for 30 training images.

	1	2	4	8	12	16	20	Avg
Niemeijer et al. [10]	0.072	0.087	0.101	0.121	0.130	0.185	0.210	0.130
Zhang et al. [17]	0.127	0.150	0.197	0.289	0.310	0.316	0.330	0.255
Javidi et al. [18]	0.130	0.147	0.209	0.287	0.319	0.353	0.383	0.261
Proposed method	0.128	0.151	0.250	0.300	0.356	0.381	0.432	0.285
